# Improved image quality of temporal bone CT with an ultrahigh-resolution CT scanner: clinical pilot studies

**DOI:** 10.1007/s11604-020-00987-5

**Published:** 2020-05-11

**Authors:** Arisa Ohara, Haruhiko Machida, Hisae Shiga, Wataru Yamamura, Kenichi Yokoyama

**Affiliations:** 1 Department of Radiology, Kyorin University Faculty of Medicine, 6-20-2, Shinkawa, Mitaka-shi, Tokyo 181-8611 Japan; 2 Department of Radiology, Kyorin University Hospital, 6-20-2, Shinkawa, Mitaka-shi, Tokyo 181-8611 Japan

**Keywords:** Ultrahigh-resolution CT, Temporal bone CT, Image quality

## Abstract

**Purpose:**

Ultrahigh-resolution CT (UHRCT) with slice collimation of 0.25 mm × 160 and matrix size of 1024 × 1024 has become clinically available. We compared the image quality of temporal bone CT (TBCT) between UHRCT and conventional multidetector CT (MDCT).

**Materials and methods:**

We retrospectively enrolled 20 patients who underwent TBCT by MDCT (matrix size, 512 × 512) and subsequently by UHRCT (matrix size, 1024 × 1024). Two independent reviewers subjectively graded delineation of normal stapes, oval window, facial nerve canal, incudostapedial joint, and tympanic tegmen. We also quantified image noise in the cerebellar hemisphere. Between MDCT and UHRCT, we compared mean subjective grades using the Wilcoxon signed-rank test and the image noise using paired *t* test.

**Results:**

Grades were significantly higher with UHRCT than with MDCT for all the anatomies (*P* < 0.001), whereas noise was significantly higher with UHRCT than with MDCT (*P* = 0.002).

**Conclusion:**

For TBCT, UHRCT shows better delineation of the fine anatomical structures compared with MDCT.

## Introduction

Multidetector row CT (MDCT) is a major diagnostic tool in temporal bone imaging but is sometimes limited in the delineation of fine and complex anatomical structures of the middle and inner ears [1–4]. Since March 2017, a state-of-the-art ultrahigh-resolution CT (UHRCT) scanner has been clinically available to improve in- and through-plane spatial resolution of CT images. Major features of this CT scanner include an improved detector system (minimal slice thickness, 0.25 mm; maximal channel number, 1792) and x-ray focus (smallest size, 0.4 × 0.5 mm) compared with conventional MDCT scanners. Whereas UHRCT has been reported to be clinically useful in various CT examinations, including chest CT [5, 6], coronary CT angiography [7–9], and CT of the artery of Adamkiewicz [10], few reports appear to have examined temporal bone CT [11]. Yamashita et al. [11] previously compared the delineation of fine anatomical structures on temporal bone CT between UHRCT with a 512-matrix size and conventional MDCT. A greater matrix size can improve microstructure delineation. We thus compared image qualities of temporal bone CT between UHRCT with a 1024-matrix size and conventional MDCT.

## Materials and methods

### Patient population

We retrospectively enrolled 132 consecutive patients who underwent routine non-contrast temporal bone CT with the UHRCT scanner between April 1, 2017 and October 31, 2017 as a follow-up study, and who had undergone CT of the same region using a conventional 64-detector CT scanner previously at our institution without any significant interval change in clinical status based on their medical records. When two or more examinations were performed with the conventional MDCT scanner, only those CT images acquired at the most recent examination were used for this study. An experienced head-and-neck radiologist with 15 years of clinical experience reviewed the axial and MPR coronal temporal bone CT images on both sides and finally included 20 non-traumatic patients (12 men, 8 women; mean age, 62 ± 23 years; age range, 11–85 years) who showed normal temporal bone by both UHRCT and conventional MDCT without intervening change on at least one side. Median interval between the two CT examinations was 13.5 months (range, 5–40 months).

This study was approved by the institutional review board at our institution. Informed consent was obtained from every patient.

### CT data acquisition

We performed temporal bone CT with both the UHRCT (Aquilion Precision; Canon Medical Systems, Otawara, Tochigi, Japan) and conventional MDCT scanners (Aquilion 64; Canon Medical Systems) using a standard helical scan method, as described elsewhere [12–14]. Scan parameters for UHRCT were: tube voltage, 120 kVp; noise index, 6.5 Hounsfield Units for 2-mm thickness with filtered back-projection by automatic exposure control; number of channels, 1792; slice collimation, 0.25 mm × 160 rows; pitch factor, 0.569; rotation time, 0.75 s; and x-ray focus size, 0.4 × 0.5 mm. Scan parameters for conventional MDCT were: tube voltage, 120 kVp; tube current, 250 mA; number of channels, 896; slice collimation, 0.5 mm × 4 rows; pitch factor, 0.625; rotation time, 0.75 s; and x-ray focus size, 0.9 × 0.8 mm. With this MDCT scanner, we fixed the tube current because automatic exposure control was unavailable; and used only the central 4 rows of detectors to reduce the effect of cone angle and thus applied quarter-offset reconstruction to improve in-plane spatial resolution.

We recorded the volume CT dose index (measured in mGy) displayed on the dose report on the CT scanner for each patient and calculated the mean volume CT dose index in our patients.

### Image reconstruction and display

For each patient, we used filtered back-projection to reconstruct temporal bone CT axial and MPR coronal images by both UHRCT and conventional MDCT using the following parameters: FOV, 10 cm; matrix size, 1024 × 1024; slice thickness and interval, 0.6 mm; and bone kernel, FC 80 for the UHRCT; FOV, 10 cm; matrix size, 512 × 512; slice thickness and interval, 0.6 mm; and bone kernel, FC 80 for the conventional MDCT. All CT images were transferred to a PACS (SYNAPSE; FUJIFILM Medical, Tokyo, Japan) and displayed with a window width of 4000 Hounsfield Units and window level of 600 Hounsfield Units.

### Image quality assessment

Two independent radiologists with 15 (Reviewer 1) and 5 (Reviewer 2) years of clinical experience freely scrolled the temporal bone CT images on the PACS using a paging method and subjectively graded the delineation of the following high-contrast structures only on the normal side in each patient: the stapes, oval window, and tympanic segment of the facial nerve canal on axial images, and the incudostapedial joint, oval window, and tympanic tegmen on MPR coronal images. We used a 5-point scale for grading: one (poor), not visible; two (fair), partially visible; three (moderate), generally visible with slight blurring or discontinuity; four (good), clearly visible with complete continuity; and five (excellent), very clearly visible with complete continuity. These anatomical structures were selected as clinically critical, but often difficult to completely and clearly delineate with a conventional MDCT scanner. Dehiscence is known to be identified in the tympanic segment of the facial nerve canal (mostly around the oval window) and the tympanic tegmen even in normal cases [15–17]. We thus defined a subjective grade of three points or less as the presence of dehiscence in these two structures.

For quantitative assessment of image noise, we placed a circular region of interest of approximately 10 mm^2^ in the cerebellar hemisphere on the same side to measure the standard deviation of the CT value as the background noise on temporal bone CT images by UHRCT and conventional MDCT in each patient.

### Statistical analysis

All continuous variables are expressed as mean ± standard deviation. We performed statistical analyses using commercially available software (IBM SPSS Statistics version 23; IBM, Armonk, NY). We used the Wilcoxon signed-rank test to compare subjective image quality graded for each anatomy on the temporal bone CT axial and MPR coronal images, the McNemar test to compare the incidence of dehiscence in the tympanic segment of the facial nerve canal and the tympanic tegmen between UHRCT and conventional MDCT. We used paired *t*-test to compare quantitative image noise after confirming the normal distribution and the Wilcoxon signed-rank test to compare the mean volume CT dose index between UHRCT and conventional MDCT. Values of *P* < 0.05 were considered statistically significant. We also used weighted kappa (*κ*) statistics to quantify inter-reviewer agreement on subjective image quality grade. Values of κ were interpreted as follows: poor, 0.00–0.20; fair, 0.21–0.40; moderate, 0.41–0.60; good, 0.61–0.80; and excellent, 0.81–1.00.

## Results

**Fig. 1:**
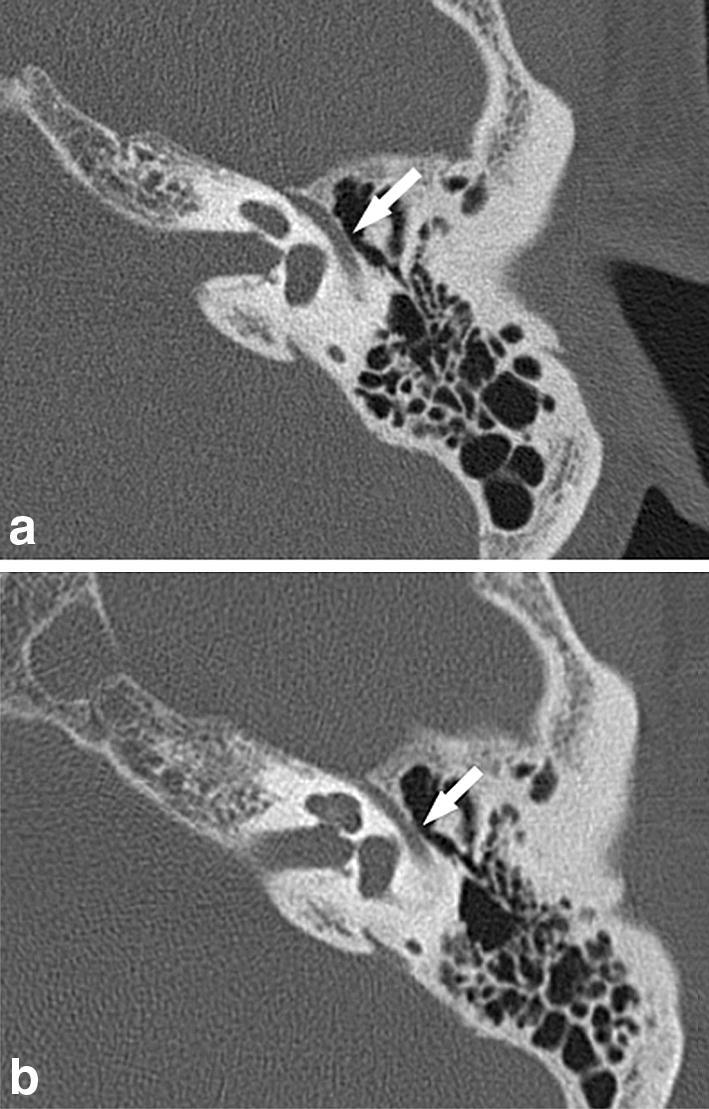
Axial images of the left temporal bone CT from the same case. Image quality scores for the tympanic segment of the facial nerve canal (arrow) by Reviewers 1 and 2 are five and four, respectively, on UHRCT (**a**), and three on MDCT (**b**) by both Reviewers. By both Reviewers, dehiscence in this anatomy was considered to be present on MDCT but not on UHRCT

**Fig. 2:**
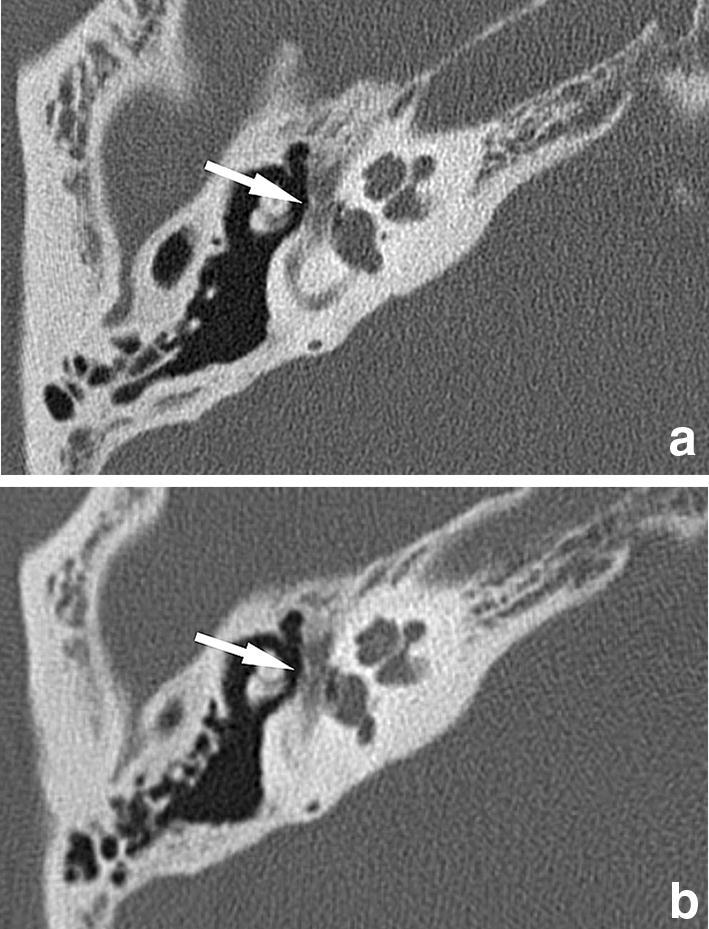
Axial images of the right temporal bone CT from the same case. Image quality scores for the tympanic segment of the facial nerve canal (arrow) by Reviewers 1 and 2 are three and four on UHRCT (**a**), respectively, and two and three on MDCT (**b**). Dehiscence in this anatomy was considered to be present by both Reviewers on MDCT; only by Reviewer 1 but not by Reviewer 2 on UHRCT

**Fig. 3:**
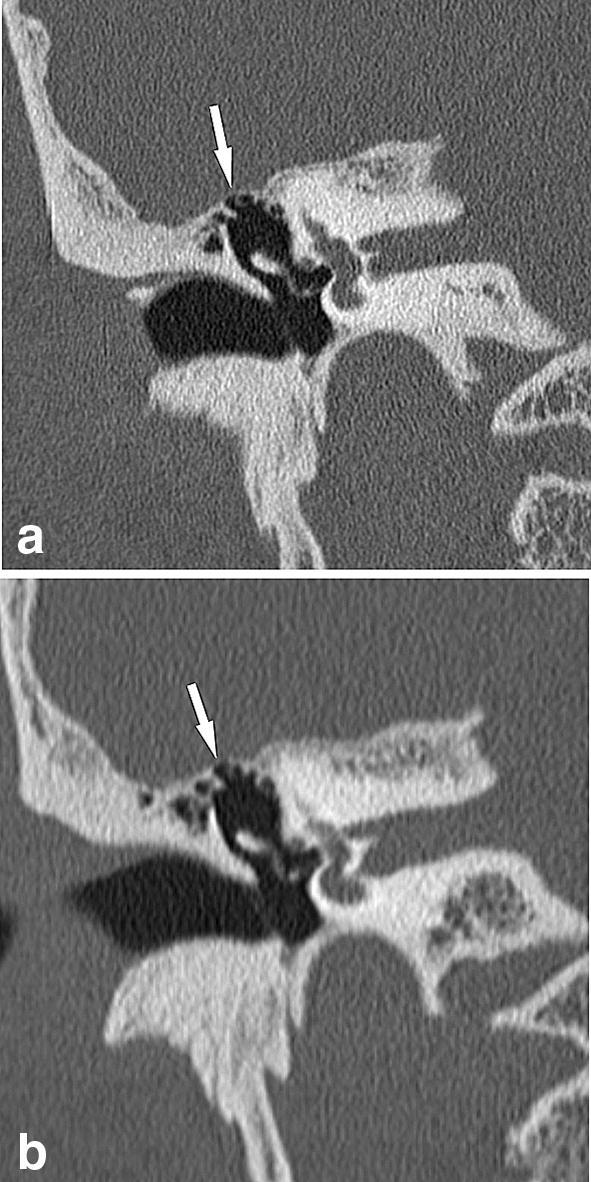
Coronal images of the right temporal bone CT from the same case. Image quality scores for the tympanic tegmen (arrow) are five on UHRCT (**a**), and three on MDCT (**b**) by both Reviewers. By both Reviewers, dehiscence in this anatomy was considered to be present on MDCT but not on UHRCT

**Fig. 4:**
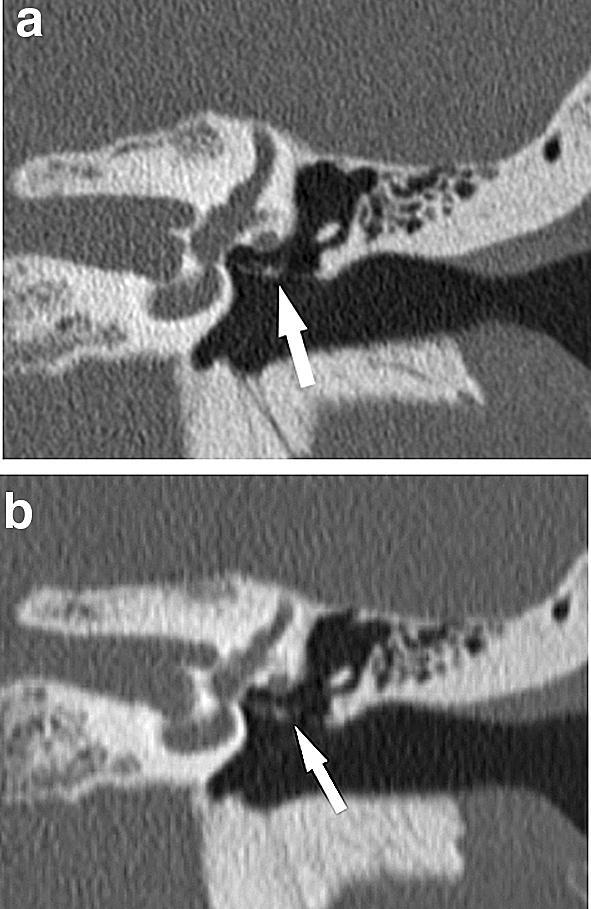
Coronal images of the left temporal bone CT from the same case. Image quality scores of the incudostapedial joint (arrow) are five on UHRCT (**a**), and three on MDCT (**b**) by both Reviewers

Table 1 shows average image quality grades for each anatomy by UHRCT and conventional MDCT by the two Reviewers. The grades on both axial and MPR coronal images were significantly better by UHRCT than by conventional MDCT for all the anatomies (*P* < 0.001) (Figs. 1, 2, 3, 4).

**Table 1:** Average image quality grades by Reviewers 1 and 2

	UHRCT	MDCT	*P* value
Axial			
Stapes	4.8 ± 0.4	3.6 ± 0.5	< 0.001
Oval window	4.7 ± 0.5	3.6 ± 0.6	< 0.001
Facial nerve canal	4.0 ± 0.8	3.2 ± 0.6	< 0.001
Coronal			
Incudostapedial joint	4.8 ± 0.4	3.6 ± 0.5	< 0.001
Oval window	4.8 ± 0.4	3.2 ± 0.4	< 0.001
Tympanic tegmen	4.9 ± 0.3	3.9 ± 0.7	< 0.001

*MDCT* multidetector row CT, *UHRCT* ultrahigh-resolution CT

The incidence of dehiscence in the tympanic segment of the facial nerve canal and tympanic tegmen was comparable between UHRCT and conventional MDCT for Reviewer 1 (*P* = 0.182 and 0.133, respectively), although that in both anatomies was significantly lower by UHRCT than by conventional MDCT for Reviewer 2 (*P* < 0.001 and = 0.023, respectively) (Table 2; Figs. 1, 2, 3).

**Table 2:** Presence of dehiscence on CT

Reviewer 1	UHRCT	MDCT	*P* value
Facial nerve canal	7/20 (35%)	12/20 (60%)	0.182
Tympanic tegmen	0/20 (0%)	4/20 (20%)	0.133

Reviewer 2	UHRCT	MDCT	*P* value
Facial nerve canal	5/20 (25%)	19/20 (95%)	< 0.001
Tympanic tegmen	0/20 (0%)	7/20 (35%)	0.023

*MDCT* multidetector row CT, *UHRCT* ultrahigh-resolution CT

Inter-reviewer agreement of all subjective image quality scores was good (*κ* = 0.69).

Mean volume CT dose index was significantly lower with UHRCT (45.3 ± 2.1 mGy) than with conventional MDCT (128.2 ± 9.9 mGy; *P* < 0.001).

Quantitative noise was significantly higher by UHRCT (151.7 ± 26.2 Hounsfield Units) than by conventional MDCT (126.9 ± 17.6 Hounsfield Units; *P* = 0.002).

## Discussion

UHRCT can improve in- and through-plane spatial resolution compared with conventional MDCT and achieve the maximal spatial resolution of approximately 0.15 mm, mainly due to the improved detector system and x-ray focus [5, 6]. In the present study, use of UHRCT significantly improved delineation of all high-contrast, fine, and critical anatomical structures on temporal bone CT compared with conventional MDCT. Yamashita et al. [11] demonstrated that only the stapedius tendon was more clearly visualized on temporal bone CT by UHRCT than by 320-detector CT. However, all other anatomical structures were similarly visualized between both, possibly due to the use of a 512-matrix size also for UHRCT, different from the situation in our study (i.e., 1024-matrix size).

Dehiscence is known to be identified in the facial nerve canal and tympanic tegmen even in normal cases [15–17]. The incidence of dehiscence of both the facial nerve canal and tympanic tegmen was higher by conventional MDCT than by UHRCT, although not significantly for Reviewer 1, possibly resulting from a greater partial volume effect due to lower spatial resolution in conventional MDCT. According to published data from cadaveric and surgical studies [15, 18–21], the incidence of facial nerve canal dehiscence ranged from 25 to 74%. According to published data from cadaveric studies, the incidence of tympanic tegmen dehiscence was reported as 20% [16, 17]. Thus, dehiscence of both the facial nerve canal and tympanic tegmen might have been overestimated by Reviewer 2 using conventional MDCT alone, as that Reviewer had less experience with temporal bone CT interpretation.

We performed helical scanning and reconstructed 0.6-mm-slice images by filtered back-projection as per our clinical routine examinations. Use of axial or volume scanning for decreasing artifacts related to helical scanning and a thinner slice thickness for improving spatial resolution, if necessary, without increasing image noise by hybrid or model-based iterative reconstruction can not only further improve delineation of the aforementioned structures and diagnostic accuracy of the dehiscence of both the anatomies, but can also achieve visualization of more subtle structures such as mucosal folds of the middle ear.

Mean volume CT dose index by conventional MDCT without using automatic exposure control (tube current-exposure time product: 187.5 mAs) was lower than that in previous studies [1, 11], but significantly higher than that by UHRCT in our study. Mean volume CT dose index by conventional MDCT was higher, but that by UHRCT was lower than the diagnostic reference levels for head CT (56–85 mGy) in the United States, European countries, and Japan [22–24]. Use of axial or volume scanning and hybrid or model-based iterative reconstruction may further reasonably reduce radiation dose by UHRCT. Whereas quantitative image noise was greater by UHRCT with better spatial resolution than by conventional MDCT, the use of the iterative reconstruction can also reduce the noise.

Our study was limited in the retrospective design and inclusion of only a small study population from a single institution. We only used the single scan and reconstruction protocol for temporal bone CT. Use of different scan and reconstruction protocols may affect the study results. The diagnosis of dehiscence of the facial nerve canal and the tympanic tegmen by temporal bone CT was not surgically confirmed. Intervals between conventional MDCT and UHRCT examinations were long (median, 13.5 months) due to the retrospective nature of the study, although shorter-interval examinations would not be practical due to the increased radiation exposure. Although we only evaluated the delineation of normal anatomical structures on temporal bone CT, further studies are needed to assess diagnostic performance for middle ear diseases such as cholesteatoma and otosclerosis. However, a comparison of diagnostic performance between conventional MDCT and UHRCT in the same patients is challenging, as these diseases are likely to change over time.

In conclusion, the latest UHRCT increases image noise but appears useful by offering sharper, clearer, more continuous delineation of fine anatomical structures in routine temporal bone CT scanning with higher spatial resolution compared with conventional MDCT. These benefits are mainly due to the improved detector system and x-ray focus.

## Abbreviations

MDCT: Multidetector CT
UHRCT: Ultrahigh-resolution CT
